# Tuning oxygen vacancy photoluminescence in monoclinic Y_2_WO_6_ by selectively occupying yttrium sites using lanthanum

**DOI:** 10.1038/srep09443

**Published:** 2015-03-30

**Authors:** Bangfu Ding, Chao Han, Lirong Zheng, Junying Zhang, Rongming Wang, Zilong Tang

**Affiliations:** 1 Key Laboratory of Micro-nano Measurement, Manipulation and Physics (Ministry of Education), Department of Physics, Beihang University, Beijing 100191, China; 2 Beijing Synchrotron Radiation Facility, Institute of high Energy Physics, Chinese Academy of Sciences, Beijing 100049, China; 3 School of Mathematics and Physics, University of Science and Technology Beijing, Beijing 100083, China; 4 State Key Laboratory of New Ceramic and Fine Processing, Tsinghua University, Beijing 100084, China

## Abstract

The effect of isovalent lanthanum (La) doping on the monoclinic Y_2_WO_6_ photoluminescence was studied. Introducing the non-activated La^3+^ into Y_2_WO_6_ brings new excitation bands from violet to visible regions and strong near-infrared emission, while the bands position and intensity depend on the doping concentration. It is interesting to find that doping La^3+^ into Y_2_WO_6_ promotes the oxygen vacancy formation according to the first-principle calculation, Raman spectrum, and synchrotron radiation analysis. Through the Rietveld refinement and X-ray photoelectron spectroscopy results, La^3+^ is found to mainly occupy the Y2 (2f) site in low-concentration doped samples. With increasing doping concentration, the La^3+^ occupation number at the Y3 (4g) site increases faster than those at the Y1 (2e) and Y2 (2f) sites. When La^3+^ occupies different Y sites, the localized energy states caused by the oxygen vacancy pair change their position in the forbidden band, inducing the variation of the excitation and emission bands. This research proposes a feasible method to tune the oxygen vacancy emission, eliminating the challenge of precisely controlling the calcination atmosphere.

Tungstates are a kind of self-activated luminescence materials. That can be divided into several categories, normal metal tungstates (MWO_4_), rare earth tungstates (Re_2_WO_6_) and poly-tungstates^1^,^2^,^3^. Since Kroger concluded that the lattice group (WO_4_^2−^/WO_6_^6−^) itself was responsible for the luminescence origin^4^, what influenced tungstates luminescence properties was explored extensively such as morphology, size and dimension^5^,^6^. In addition to intrinsic emission of anion-cation groups, there was also emission from defect states, inevitably incurred because of the abundant synthesis methods and flexible annealing temperatures and atmospheres^7^,^8^,^9^. For example, the photoluminescence intensity of amorphous BaWO_4_ was higher than that of crystalline BaWO_4_ because of different annealing temperatures^10^. Therefore, the tungstate hosts luminescent properties were of great interest^11^,^12^.

Though various methods have been employed to improve luminescent properties, process parameters especially annealing atmospheres, in particular oxygen partial pressure, were not controlled precisely^13^. An easy and convenient approach is to dope impurities in matrixes to enhance emission or obtain multi-color emission^14^,^15^,^16^. The impurity can be any elements for the non-isovalent doping, such as trivalent rare earth and monovalent alkaline metal ions^17^,^18^. This method has been extensively investigated in luminescent compounds, photo-catalysts, and magnetic materials. For example, La^3+^-doped ZnO has high photocatalytic activity^19^, and LaCoMnO_6_ presents the coexistence of ferromagnetic and antiferromagnetic properties with increased Ca^2+^ substitution amounts^20^. For tungstates, this approach mainly aims at enhancing luminescent intensity, changing optical activity, or broadening emission wavelength range^21^,^22^,^23^. For instance, the emission wavelength of CaW(Mo)O_4_ nanoparticles was tuned from blue-green or yellow to white by increasing the Dy^3+^ concentration^24^. In addition, there are considerable reports on the La^3+^ doping effect on the luminescence properties of PbWO_4_^25^,^26^. According to the first principles study, different electronic compensation effects lead to different defect states in the band gap for high and low doping conditions, which explains the 420 nm band origin and new red absorption band^27^.

Similarly, the isovalent doping can also change the luminescence properties of matrixes such as salt compounds and oxides^28^,^29^. White up-conversion luminescence and enhanced emission were obtained in Yb^3+^/Er^3+^/Tm^3+^ doped YAlO_3_ and Ca^2+^-doped MgO nanocrystals. The isovalent doping technique is often considered in tungstates, especially rare earth tungstates. The energy transfer processes of rare earth tungstates doped with Eu^3+^, Sm^3+^, Dy^3+^ and co-doped with Eu^3+^/Tb^3+^ have been summarized by Kaczmarek and Deum^30^. However, the photoluminescence mechanism of the matrix such as lanthanum, lutetium and yttrium tungstates (La_2_WO_6_, Lu_2_WO_6_, Nd_2_WO_6_ and Y_2_WO_6_)^31^,^32^,^33^,^34^,^35^,^36^,^37^ after importing isovalent and non-activated ions is still open to be exploited. The luminescent properties of Bi_2_WO_6_ with and without La^3+^ doping were compared at low temperature 4 K^38^, having found that La^3+^ doping increased the stokes shift of the matrix luminescence.

Various activators and sensitizers have been doped to improve the emission of monoclinic Y_2_WO_6_^33^,^34^,^35^,^36^,^37^,^39^. The main purposes of these investigations are how to obtain white-light emission or promote energy conversion efficiency. The oxygen vacancy and local crystal structural regulation of monoclinic Y_2_WO_6_ by non-activated ions have not been reported until now. Recently, we^40^ found that the atmosphere and calcination temperature induced the changes of oxygen vacancy concentration and tungsten coordination number in monoclinic Y_2_WO_6_, and thus affected the appearance of long-wave excitation and near-infrared emission bands. By calcining Y_2_WO_6_ in the air at 1200°C, the 340 nm excitation band, caused by low-concentration oxygen-vacancy, was substantially enhanced in comparison with those calcined at high temperature or in argon. Calcining in argon resulted in strong infrared emission because of the increased oxygen vacancy concentration^40^. But the oxygen partial pressure, which depends on the airtightness of the furnace, cannot be controlled purposefully when the sample is calcined in the air. Therefore, it is ideal to tune the oxygen vacancy by a simple and feasible method on the basis of the doping approach advantages. Moreover, the mechanism behind the impurity effect on the luminescence of the matrix needs more intensive investigation to make up for the deficiency of the previous theory.

In this paper, a series of Y_2_WO_6_:xLa^3+^(x = 0 and 0.01–0.05) powders are synthesized in air condition at 1250°C through the simple solid phase reaction. These samples show strong visible emission. It is surprising to find that the 340 nm excitation intensity of the powders with dopant concentration not more than 3 at% is stronger than that of the pristine Y_2_WO_6_. The La^3+^ doping can also produce many new excitation bands in the ultraviolet and visible regions. These new excitation bands are ascribed to the different oxygen vacancy pair behavior induced by occupation variation of La^3+^ in the three Y sites. When La^3+^ enters into the Y2 (2f) site at low concentration, the oxygen vacancy pair energy band locates just above the valence band (VB), intensifying the 340 nm excitation band. At high doping concentration, the occupation number of La^3+^ in the Y3 (4g) site becomes high, bringing new localized energy states and excitation bands and weakening the 340 nm excitation intensity. The change of oxygen vacancy energy states generates different luminescence phenomenon.

## Results and Discussion

### Crystal structure

**Figure 1:**
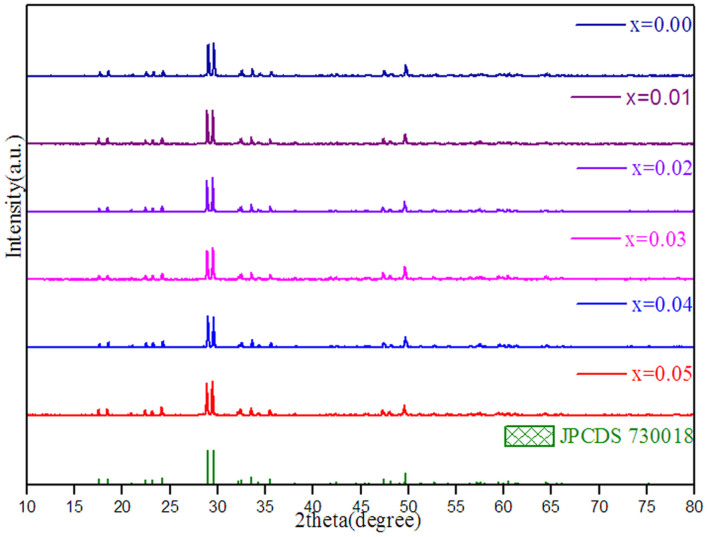
XRD patterns of Y_2_WO_6_:xLa^3+^(x = 0 and 0.01–0.05) powders calcined at 1250°C under air condition and PDF card 73-0018.

The crystal structure of pure Y_2_WO_6_ is monoclinic phase with space group 13-P_12_/C_1_-C_2h_^4^ reported by Efremov^41^, whose inorganic crystal structure database (ICSD) number is 20955. In order to check the phase purity of as-prepared samples, X-ray diffraction (XRD) measurement results are plotted in Figure 1. All the XRD patterns agree well with the patterns of powder diffraction file (PDF) card 73-0118 and no peaks from other phases such as La_2_WO_6_ are observed. Due to the effective ion radius difference of Y^3+^ (0.96 Å and 1.019 Å for VII and VIII coordination) and La^3+^ (1.10 Å and 1.160 Å for VII and VIII coordination)^42^, the diffraction peaks of the La^3+^-doped samples slightly deviate when La^3+^ substitutes for Y^3+^.

The structure refinement of Y_2_WO_6_:xLa^3+^(x = 0.03, 0.05) powders was performed through the general structure analysis system (GSAS) software package^43^. The calculated patterns are consistent with the experimental XRD patterns (Supporting Information, Figure S1(a) and (b)). The atomic positions, occupation numbers, crystal structure and refinement parameters are listed in Table 1. The volumes of the samples gradually become larger with increasing La^3+^ content, while the atomic coordination and lattice parameters change a little. From the occupation numbers, one can see that La^3+^ enters into three Y sites simultaneously at any concentrations, occupying mainly the Y2 (2f) and Y3 (4g) sites and minorly the Y1 (2e) site. In 3.0 at% and 5.0 at% La^3+^-doped samples, the occupation numbers of La^3+^ at Y1 (2e), Y2 (2f), and Y3 (4g) sites are 0.009, 0.035, and 0.02 and 0.01, 0.052 and 0.044, respectively. Hence, the occupation numbers of La^3+^ in the Y2 (2f) and Y3 (4g) sites gradually become higher with increasing La^3+^ concentration, and their variation quantities are 0.017 and 0.024, whereas, La^3+^ hardly enters into the Y1 (2e) site under this experimental condition.

There is a W (4g) site possessing C_1_ symmetry, which is surrounded by six O atoms to form distorted octahedral coordination. The bond lengths of the six W-O are not identical each other. Three kinds of Y sites were coordinated with eight (2e, 2f) and seven (4g) oxygen atoms constructing polyhedron coordination. The Y1 (2e) and Y2 (2f) sites have C_2_ symmetry, while the Y3 (4g) site takes on C_1_ symmetry^44^. The average Y-O bonds length is 2.3 Å for three Y sites. According to Pauling's electrostatic valence rule^45^, the bond strengths (S) of yttrium and tungsten are S_Y1-O_ = 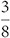, S_Y2-O_ = 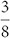, S_Y3-O_ = 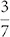, and S_W-O_ = 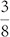. Thus the sum of ζ (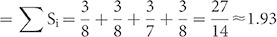) is approximately equal to the O^2−^ valence, indicating that Y_2_WO_6_ has stable structure. Because of isoelectrons between La^3+^ and Y^3+^, Y_2_WO_6_:xLa^3+^ also possesses a stable structure as evidenced by the invariability of the XRD patterns (Figure 1)^46^,^47^,^48^,^49^.

### Photoluminescence properties

**Figure 2:**
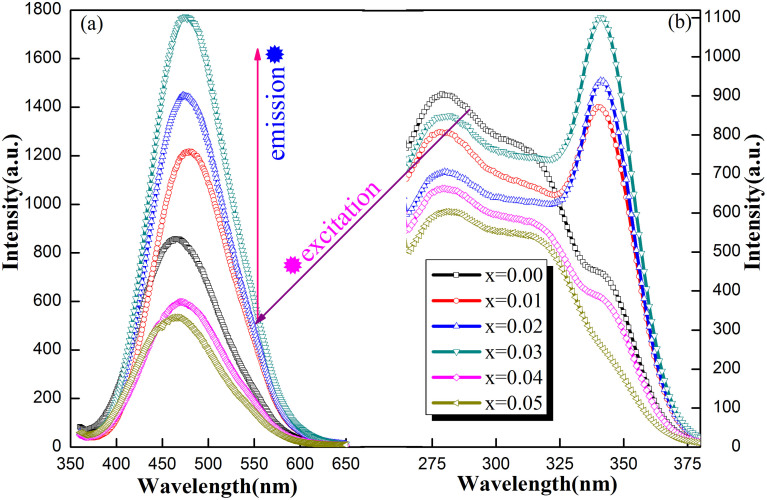
PL emissions (a) and excitations (b) of Y_2_WO_6_:xLa^3+^ (x = 0 and 0.01–0.05) powders calcined at 1250°C in air.

Figure 2 shows the photoluminescence (PL) emission and excitation spectra of all Y_2_WO_6_:xLa^3+^(x = 0 and 0.01–0.05) samples. Under 340 nm excitation, the PL emission spectra have broad band shapes covering from 365 to 650 nm with maximal value around 470 nm. This band results from charge transition emission between the local oxygen 2p states (just above the VB) and the conduction band (CB)^40^. The PL intensities in pure and low-concentration La^3+^-doped samples (x ≤ 0.03) are stronger than those of high-concentration La^3+^-doped powders. From Figure 2(a), the asymmetric shapes of emission spectra do not depend on dopant concentration and remain single-peak frameworks. In addition, the total, low-energy side, high-energy side half widths and peak values show slight dependence on La^3+^ concentration (Supporting Information, Table S1)^50^. Thus, the emission band is composed of at least two overlapping bands^50^.

**Figure 3:**
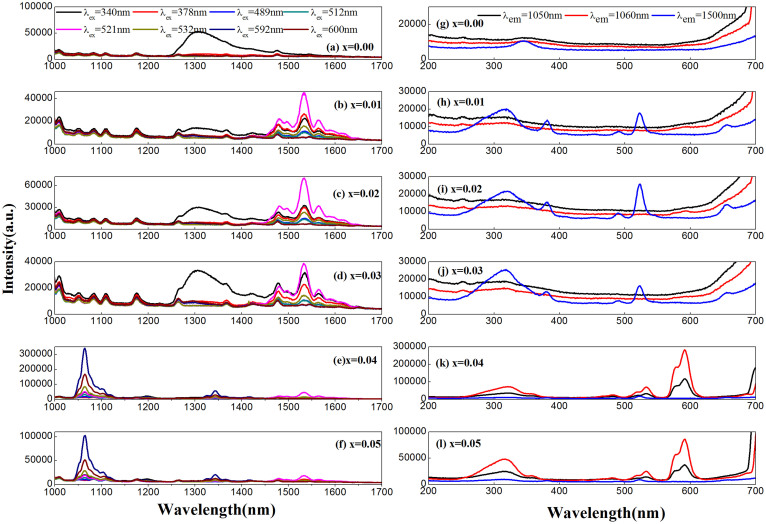
(a)–(f) Emission spectra of Y_2_WO_6_:xLa^3+^ phosphors burning in air conditions measured under different excitation wavelength. (g)–(l) Excitation spectra of Y_2_WO_6_:xLa^3+^ powders measured by monitoring emission in the near-infrared region.

Moreover, the powders also show the near-infrared emission in the scope of 1000–1700 nm as depicted in Figure 3(a)–(f). The pure sample displays strong luminescence in the 1300–1400 nm region and weak emission in the 1450–1650 nm region. Under the 340, 378, 489, 512, 521, and 532 nm excitations, the 1–3 at% La^3+^-doped samples show strong emissions in two regions (1300–1400 nm and 1450–1650 nm). When the concentration continues to rise to 4 at% and 5 at%, the infrared luminescence is also observed in two regions (1300–1400 nm and 1450–1700 nm), besides much stronger emission from 1000 to 1150 nm. In order to exhibit the fine structure clearly, we have enlarged the emission spectra of 4 at% and 5 at% La^3+^ doped samples from 1300 nm to 1700 nm (supporting information, Figure S2). Tuning the La^3+^ content changes the infrared luminescence, suggesting the presence of ample local states in the band gap^51^.

To obtain a better understanding of photoluminescence phenomenon, Figure 2(b) displays the excitation spectra of all samples by monitoring 520 nm emission. All samples show three excitation bands containing two short wavelength bands (peaking at 280 and 310 nm) and one long wavelength band (peaking at 340 nm). When La^3+^ doping concentration increases from 1 at% to 3 at%, the 340 nm band gradually intensifies compared with the pristine sample. When the doping concentration exceeds 3 at%, the intensity of this band gradually weakens. A similar excitation band also appeared in other tungstates such as CaWO_4_^52^ and ZnWO_4_:Bi^3+^, Eu^3+^ phosphors^53^. Their origins were ascribed to oxygen vacancy and ^1^S_0_ → ^3^P_1_ transitions of Bi^3+^. For air-annealed Y_2_WO_6_ samples^34^,^40^, this band was originated from low-concentration oxygen vacancy. Therefore, the dramatic variation of the 340 nm excitation band intensity undoubtedly originates from the oxygen vacancy defect and La^3+^ doping effect. The tunable defect emission intensity is obtained by changing the La^3+^ content. When the detector wavelength extends to the near-infrared ranges, many new excitation peaks, such as 380, 491, and 523 nm, appear in 1–3 at% La^3+^-doped powders. For samples doped with higher content of La^3+^, a series of peaks at 380, 482, 522, 533, 577, and 591 nm are observed. These new excitation bands were ascribed to the oxygen vacancy pair in Y_2_WO_6_^40^. In the pristine sample, only the 340 nm excitation band is observed. Hence the behavior of oxygen vacancy changes a lot due to the incorporation of La^3+^ in Y_2_WO_6_.

### Local crystal environments

In order to explore these new excitation bands origins and intensity variation of the 340 nm excitation band, we carried out Raman measurement to determine the local vibration structure of all the samples. According to group theory^40^,^54^, Y_2_WO_6_ crystals have 3N = 3 × 36 = 108 distinct Raman and Infrared vibration modes. As we know, Raman spectra of tungstates can be identified with two types of groups as external and internal vibration modes^55^. The external vibration modes concerning lattice phonons correspond to the motion of polyhedral YO_8/7_ clusters. The internal vibration modes originates from the vibration of distorted octahedral WO_6_ clusters^56^,^57^, which are written as 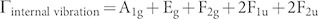.

The Raman active vibration modes of all Y_2_WO_6_:xLa^3+^ powders ranging from 100 to 1000 cm^−1^ are shown in the Supporting Information (Figure S3). Table 2 lists all the Raman peaks positions and the coordination numbers according to similar chemical formulas M_2_W(Mo)O_6_(M = La, Nd, Sm, and Bi)^57^. In the undoped sample, the strongest peak at 834 cm^−1^ is assigned to the W = O symmetrical stretching vibration A_1g_ mode and has a half width of 20 cm^−1^, which indicates that the coordination number (CN) between tungsten and oxygen is 6^40^,^57^. The weak peaks at 707, 694, 668, 645, 621, 596, 550, 521, 501, 471, 446, 426, 394, 375, 365, 354, 340, 309, 290, 282, 270, 253, 238, 225, 215, 199, 192, 178, 142, 127, 118, and 104 cm^−1^ are internal vibration modes representing the tungsten CN being 6. Moreover, there are a small amount of five coordination tungsten atoms with the peaks position at 170 and 155 cm^−1^. Compared with the previous results of Y_2_WO_6_ calcined in air^40^, it is confirmed that the local crystal structure is sensitively affected by the calcination environment which depends on the individual furnace used during the samples preparation.

The Raman spectra of La^3+^-doped samples differ from that of the pure sample by exhibiting some new Raman peaks. In Y_2_WO_6_:xLa^3+^(x = 0.01–0.03), the peaks at 798, 773, 381, and 207 cm^−1^ suggest that some tungsten atoms have tetrahedral coordination because the peaks positions are similar to those of La_2_MoO_6_, Bi_2_MoO_6_ and Nd_2_MoO_6_^57^. The peak value 156 cm^−1^ is analogous to that of Sm_2_WO_6_, which indicates that CN of several tungsten atoms reduces to 5. Therefore, there exist tetrahedral and pentacoordinated tungsten atoms in 1–3 at% La^3+^-doped samples. For high-concentration La^3+^-doped samples, the peak shapes and numbers are almost similar to those of 1–3 at% La^3+^-doped samples. In addition, the sudden enhanced peak at 934 cm^−1^ may be a combined tone with the sum of 773 and 156 cm^−1^. Therefore, the tetra- and penta-fold tungsten atom numbers become larger after La ions are introduced into Y_2_WO_6_. To study the crystal environments further, the extended X-ray absorption fine structure (EXAFS) measurements of W-L_III_ absorption in Y_2_WO_6_:xLa^3+^(x = 0 and 0.01–0.05) samples are applied to determine the local structure around W atoms. Through Fourier transformation of the fine structure signals, Figure S4 shows the radical structure functions of W atoms. A strong peak in Figure S4 corresponds to the nearest neighbor O atoms of W ion. Furthermore, the fitting results are given in Table S2 by further Fourier fitting of this peak.

The average coordination number (CN) of tungsten and oxygen can be calculated as CN = (attenuation factor) × 6/A, where A is the value of W-O in standard sample (generally 0.7–0.9) and 6 is the theoretical coordination number. From the table S2, the average number of tungsten is reduced with the increase of La^3+^ content. Hence, it is confirmed further that oxygen vacancy concentration becomes high gradually. Though the attenuation factor of 2 at% La^3+^ -doped sample is slightly larger than those of 1 at% and 3 at% La-doped samples, the three values are assumed approximately equal. The tungsten CN is gradually reduced with increasing La^3+^ concentration, which hints that oxygen vacancies exist in all samples. Since the external environments are the same in the experiment, the oxygen vacancy variation is undoubtly ascribed to the doping effect of La^3+^.

**Figure 4:**
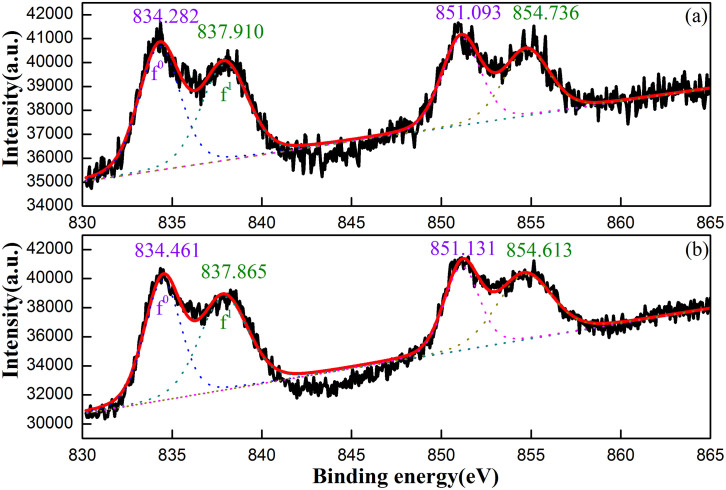
XPS and their peak fitting curves of the La 3d region for the Y_2_WO_6_:xLa^3+^ powders with x = 0.03 (a) and x = 0.05 (b).

In order to shed more lights on the La^3+^ information in Y_2_WO_6_, the XPS spectra of the La 3d core level for the Y_2_WO_6_:xLa^3+^ with x = 0.03 and 0.05 are shown in Figure 4. The La 3d XPS in the two samples can be fitted as a superposition of four Gaussian components. The peaks at 834 and 851 eV can be attributed to two spin orbits of La 3d_5/2_ and La 3d_3/2_, respectively^58^. The other two peaks at 837 and 854eV are La 3d satellite peaks. Hence, the double peak structure of each spin-orbit split agrees with the reported literatures^59^. As we know, the split spin orbit reflects states with configurations [3d^9^]^hole^4f^0^L and [3d^9^]^hole^4f^1^[L]^hole^, where L indicates the oxygen ligand. Generally, the f^0^ dominates the low binding energy signals, and the high binding energy is referred to the f^1^ peaks. Because the doping concentration has no obvious effect on the La 3d f^1^/f^0^ intensity ratio, the f^1^-f^0^ energy separation and peak shift are considered.

The f^1^-f^0^ separation values are 3.628 eV and 3.404 eV in these two samples, which differ from those in ABO_3_ perovskites, La_2_CuO_4_ (3.1 eV), LaCoO_3_ (4.3 eV) and La_1.85_Ba_0.15_CuO_4_ (5.3 eV)^59^. Thus, the equivalent doping can change the f^1^-f^0^ energy separation value, which is similar to that reported in the literature (3.6 eV for La^3+^- doped ZnO)^60^. Moreover, the La 3d_5/2_ and La 3d_3/2_ peaks shift to higher energy by 0.179 eV and 0.038 eV, and the satellite peaks shift to lower energy by 0.045 eV and 0.113 eV from x = 0.03 to x = 0.05 samples. These shifts really reflect the change in chemical potential. Different peak positions and f^1^-f^0^ energy separation values reflect different contents of La in the three Y sites^61^, which agrees with Retiveld refinement results. The La 3d_5/2_ and La 3d_3/2_ splitting distances for the x = 0.03 and x = 0.05 samples are estimated to be 16.81 and 16.670 eV, respectively, which further confirms that the occupancy site variation of La with its concentration induces the chemical state change.

### Photoluminescence mechanism

**Figure 5:**
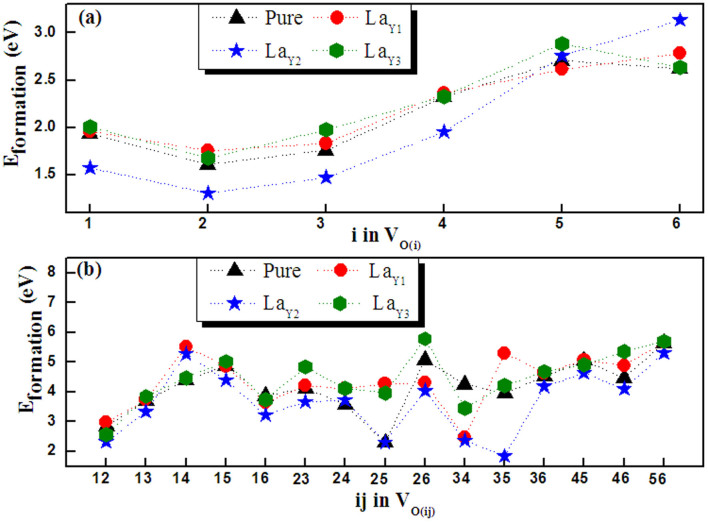
The defect formation energies of (a) V_O(i)_, V_O(i)_ + La_Yk_ (k = 1,2, and 3) and (b) V_O(ij)_, V_O(ij)_ + La_Yk_ in oxygen-rich conditions.

To obtain the deep understanding of luminescence origin, the first principle method is often applied to derive electronic structures of luminescent materials^62^. The appearance of four- and five-coordination tungsten atom numbers from the pure to different-concentration La^3+^-doped samples indicates that the oxygen vacancy concentration increases gradually with incorporation of La^3+^ into the samples. Hence, we first establish a perfect 1 × 2 × 1 Y_2_WO_6_ supercell, and then one or two oxygen atoms next to tungsten are removed to constitute single and twin oxygen vacancies together with replacing the nearest Y atom with a La atom (these models are labeled as La_Yk_ + V_O(i)_ and La_Yk_ + V_O(ij)_ with k = 1, 2, and 3 and i, j = 1, 2, 3, 4, 5, and 6, i ≠ j). Under oxygen-rich atmosphere, the defect formation energies (E_formation_) of various models when oxygen vacancy locates at different sites are plotted in Figure 5. As illustrated in Figure 5(a), for the models containing one oxygen vacancy, the variation rules of E_formation_ are the same for the undoped and La^3+^-doped models expect for the model with V_O(6)_. Their average values are calculated as 2.1593, 2.2175, 2.0334, and 2.2521 eV for pure and La^3+^-doped models when La substitutes for Y at three sites. Comparing these two average E_formation_ for V_O(i)_ and La_Y2_ + V_O(i) _models, it can be found that when La^3+^ replaces Y2, the formation energy is reduced. This indicates that La^3+^ enters into the Y2 site most easily, and doping La^3+^ into Y_2_WO_6_ can really promote the formation of oxygen vacancies.

Though the average E_formation_ for V_O(i)_ is smaller than those of La_Y1_ + V_O(i)_ and La_Y3_ + V_O(i)_, La^3+^ also can occupy Y1 and Y3 sites in the process of high-temperature calcination. Therefore, the probability of La entering into the Y2 site exceeds that of entering into Y1 and Y3 sites. The calculation results accord well with those of XRD refinement. For the La_Yk_ + V_O(ij)_ models, the minimal E_formation_ is located at different sites for the four configurations (V_O(ij)_ and La_Yk_ + V_O(ij)_). The average values of the four cases are 4.1819, 4.3838, 3.6547, and 4.4581 eV. Similarly, the E_formation_ average value of La_Y2_ + V_O(ij)_ is smaller than that of the V_O(ij)_, and the difference of average E_formation_ between V_O(ij)_ and La_Yk_ + V_O(ij)_ (k = 1 and 3) samples is very small. Hence, the La^3+^ doping induces oxygen vacancy increase, which is consistent with the analysis of Raman spectra and synchrotron radiation.

For self-activated luminescent tungstates, the CB and VB are mainly composed of W 5d and O 2p states. Thus, the tungstate luminescence origin is intrinsic luminescence^62^. Moreover, luminescence caused by intrinsic defects such as oxygen vacancies or interstitial atoms also exists in tungstates^63^,^64^. For our samples, there exists amply oxygen vacancy luminescence information. As previously reported, the origin of the 340 nm excitation band is ascribed to low-concentration oxygen vacancy namely some five coordination tungsten atoms^40^. In samples containing La^3+^, the oxygen vacancy concentration increases in comparison with the pristine Y_2_WO_6_, resulting in some four and five-fold tungsten atoms.

Based on these results, we calculate the electronic structure of the samples containing both La^3+^ and an oxygen vacancy pair, to exploit the role of La doping on the behavior of the oxygen vacancy pair. As found previously^40^, single oxygen vacancy accounts for the excitation bands shorter than 400 nm (340 nm), while oxygen vacancy pair causes a series of excitation bands from 380 to 600 nm. We only need to find out why in the low-concentration La^3+^-doped sample which contains an oxygen vacancy pair like the high-concentration La^3+^-doped samples the 340 nm excitation band dominates. La^3+^ prefers to enter into the Y2 site at low concentration and then enters into the Y3 site at high concentration. Therefore, we studied the electronic structures of samples containing an oxygen vacancy pair, where La^3+^ occupies three Y sites, namely La_Y1_, La_Y2_, and La_Y3_, respectively. When the E_formation_ of these structures are closest to the average value of E_formation_ in La_Yk_ + V_O(ij)_, we calculated their electronic properties.

On the basis of the Y_2_WO_6_ crystal structure^65^, three-type Y sites are surrounded by different kinds of oxygen atoms. The nearest neighbors of the Y1 (2e) site are O3(2), O4(2), O5(2), and O6(2) (the numbers in parentheses represent oxygen atom numbers). Similarly, O1(2), O2(2), O4(2), and O6(2) surround the Y2 (2f) site, and O1(1), O2(1), O3(1), O4(1), O5(2) and O6(1) surround the Y3 (4g) site. When La enters the Yk site, the nearest oxygen atoms escape to form oxygen vacancy easily, and then the other oxygen atoms different from the nearest oxygen species escape. Hence, the average E_formation_ can be calculated for the nearest oxygen vacancy in the La_Yk_ + V_O(ij)_ models. These average values are 4.6778, 3.7884 and 4.4581 eV for La_Y1_ + V_O(ij)_, La_Y2_ + V_O(ij)_, and La_Y3_ + V_O(ij)_, respectively. Therefore, the electronic structures of La_Y1_ + V_O(36)_, La_Y2_ + V_O(24)_, and La_Y3_ + V_O(14)_ configurations are calculated, because their E_formation_ are closest to the average E_formation_.

**Figure 6:**
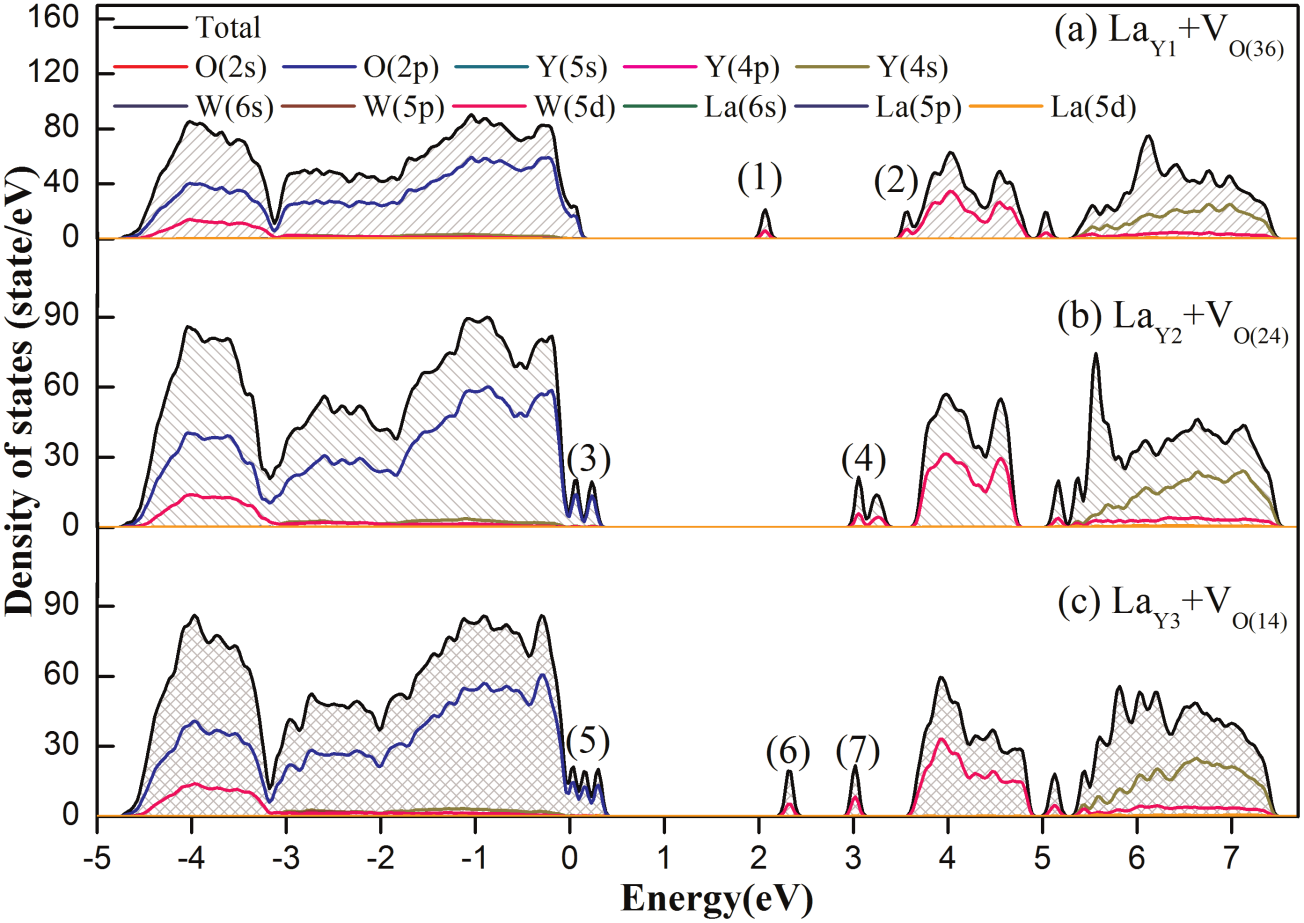
The total and partial density of states of (a) La_Y1_ + V_O(36)_ (b) La_Y2_ + V_O(24)_ and (c) La_Y3_ + V_O(14)_.

Figure 6 displays the total density of states (DOS) and partial DOS of the constituted atoms. The CB and VB are mainly composed of W 5d and O 2p states with small contributions of Y 4d. The contributions of all the La electron states for VB and CB are almost zero. The W 5p, Y 4s, W 5s, La 6s, La 4d, Y 4p, O 2s, and La 5p are located below the VB from −45 eV to −10 eV. The electronic structure properties of Y_2_WO_6_ are similar to those of some tungstates and molybdates such as scheelite CaWO_4_ and wolframite ZnMoO_4_^66^,^67^. Therefore, the luminescence origin of Y_2_WO_6_ is mainly ascribed to the charge transfer transition between W and O^68^. Moreover, the local state positions and numbers for the La_Y2_ + V_O(24)_ model are similar to those of Y_2_WO_6_ with low-concentration oxygen vacancy^40^. For La_Y1_ + V_O(36) _and La_Y3_ + V_O(14) _models, these states resemble those of Y_2_WO_6_ with high-concentration oxygen vacancy^40^. Thus, these differences of local states induce different excitation and emission phenomena. In low-concentration La^3+^-doped samples, La mainly enters into the Y2 sites resulting in ample oxygen vacancy pair. When these samples are radiated under ultraviolet (UV) light larger than the gap value (E_gap_) 3.75 eV, the electrons jump from the VB to the CB then relax and finally emit photons. Thus, the 280 nm and 310 nm excitation bands can be produced. When the UV light energy is smaller than E_gap_, the electrons in (3) states (local O 2p states) jump to the CB, generating the 340 nm excitation peak, while electrons jumping from (3) to (4) (local W 5d states) produce the 378 nm weak excitation band. Therefore, the 340 nm peak intensifies gradually with increasing La_Y2_ numbers (not more than Y2 sites numbers). When the doping concentration increases, the content variation of La_Y3_ is larger than those of La_Y2_. The electrons in (5) also jump to the CB corresponding to 340 nm excitation. However, the transition from (5) to (6) becomes a direct transition leading to strong 592 nm excitation, which weakens the electron transition for the contribution of 340 nm. Therefore, the 340 nm intensity becomes weak (does not disappear) in high-concentration La^3+^-doped samples.

From a phenomenological viewpoint, the occupation number of Y2 site becomes higher but does not reach saturation, and La^3+^ tends to occupy the Y3 sites at high doping concentrations. Thus, the 340 nm band intensity strengthens at low La content and then weakens with further increasing doping concentration. In addition, a few La^3+^ ions enter into the Y1 site as shown in table 1, and the transitions between the VB and the local states (1) can produce some excitation peaks around 500 nm. When La^3+^ occupies different Y sites, along with the oxygen pair, the local crystal structure shows different changes such as bond length and electronic density. The W-O bond length was measured as shown in Table S3. Since the six W-O bond lengths are unequal, the average values are computed. The average bond length variations in models La_Y1_ + V_O(36)_, La_Y2_ + V_O(24)_, and La_Y3_ + V_O(14)_ are 0.048, 0.026, and 0.045 Å, respectively. One can see that the bond lengths change relative to the pristine system in model La_Y2_ + V_O(24)_ is the smallest, and thus smallest distortion promotes the 340 nm excitation intensity. For models La_Y1_ + V_O(36)_ and La_Y2_ + V_O(24)_, bigger distortions lead to other forbidden transitions becoming allowed transitions, thus weakening the 340 nm intensity. Different bond length variations result in different local state distributions in the band gap.

Table 3 lists the energy level positions of VB maximum (VBM), local states, and CB minimum (CBM). From Figure 6, Figure S5 and Table 3, one can see that the 340 nm excitation band weakens or disappears when La^3+^ enters into the Y3 or Y1 sites. Therefore, the first principle calculation explains the gradual death of the 340 nm excitation band and the emergence of many new peaks. Finally, the schematic oxygen vacancy forming process is plotted (Figure S6).

### Conclusion

In the present paper, we study the concentration effect of isovalent La^3+^ doping on the photoluminescence of monoclinic Y_2_WO_6_. The incorporating of La^3+^ into the matrix favors the formation of single and coupled oxygen vacancies. At low doping concentration, La^3+^ prefers to occupy the Y2 (2f) site, while at high concentration, it mainly occupies the Y3 (4g) site. When La occupies the Y2 (2f) site, the local states caused by the oxygen vacancy pair locate just below the CBM and connect with the VBM. When La occupies the Y3 (4g) site, new local energy bands appear. As a result, besides the emission of the W-O group in the visible region, there appear new emission and excitation bands because of the mid-gap excitation using light longer than 320 nm. At low La^3+^ doping concentration, the 340 nm excitation band is substantially intensified, resulting in visible emission. A series of excitation bands in the visible region also appear, causing strong near-infrared emission. The abundant change of the excitation and emission spectra with the La doping is ascribed to the single and coupled oxygen vacancy change and the selective occupation of La to different Y sites. In our previous articles^34^, the Y_1.98_WO_6_:0.02Sm^3+^ phosphors can emit white light under 340 nm excitation. The luminescence is mainly originated from the tungstate group and Sm^3+^ emission. In this paper, La doping in monoclinic Y_2_WO_6_ greatly improves luminescence intensity under 340 nm excitation. Therefore, the strong white-light emission can be anticipated through co-doping the non-activated La^3+^ and luminous Re^3+^ (Sm^3+^, Eu^3+^) in self-activated Y_2_WO_6_ host under the near-violet irradiation.

## Experiment and calculation details

### Samples preparation and characterization

Y_2_WO_6_:xLa^3+^(x = 0 and 0.01 ~ 0.05) powders were prepared through solid-state reaction. The detailed experiment steps and characterization methods were described previously^34^,^40^. For La^3+^-doped samples, the raw materials were added different amounts of La_2_O_3_. The chemical state of the La element was examined by x-ray photoelectric spectra (XPS) using a Thermo-electron ESCALAB 250 spectrometer equipped with monochromatic Al X-ray source (1486.6 eV).

### Calculation procedures

To determine formation energy and the electronic structure, we use VASP software to simulate the doping effect in periodic supercell structures^69^. In the following, the La_Y1_ model was used an example to illustrate the calculation steps. First, a unitcell was built, and then relaxed fully. Second, the relaxed unitcell was expanded to a 1 × 2 × 1 supercell. For samples containing single or coupled oxygen vacancy, we constructed and fully optimized the 1 × 2 × 1 supercell, where one La substituted for Y1 and O1 or O1 and O2 (six-type oxygen sites) atom(s) near the W atom neighboring La was removed. Third, the defect formation energy (E_formation_) of all La_Yk_ + V_O(ij)_ models were calculated. Finally, when the E_formation_ of one model was closest to the average value of the fifteen models in every type of La_Yk_, the DOS and energy band structure were computed. In order to overcome the bandgap underestimation drawback of density function theory (DFT) calculation, the generalized gradient approximation (GGA) + U method was applied in all calculations. Through a series of tests, the optimal U values for O, Y, La and W were found to be 4.5, 0.0, 0.0 and 9.9 eV^40^.

## Author Contributions

B.F.D. completed the writing of the manuscript and first-principle calculation. C.H. performed the sample preparation and all the experimental tests. L.R.Z. fitted the data of synchrotron radiation. J.Y.Z. designed the whole research and revised the articles. R.M.W. and Z.L.T. participated in the discussion of the data and proposed many good suggestions.

## Supplementary Material

Supplementary Information Supplementary Information

## Figures and Tables

**Table 1:** Atomic positions, occupation numbers, crystalline structure, and refined parameters of La^3+^-doped Y_2_WO_6_ samples. The crystal parameters of Y_2_WO_6_ are referenced in Ref. 41

Samples		Y_2_WO_6_:0.03La^3+^				Y_2_WO_6_:0.05La^3+^				Y_2_WO_6_			
Atoms	Wyckoff	x	y	z	occupation	x	y	z	occupation	x	y	z	occupation
W	4g	0.27960	0.25318	0.38794	1.00	0.27867	0.2544	0.3879	1.00	0.2800	0.2524	0.3879	1.00
Y1	2e	0.00000	0.7360	0.25000	0.991	0.00000	0.7389	0.250000	0.99	0	0.7366	0.25	1.00
Y2	2f	0.50000	0.6857	0.25000	0.965	0.50000	0.6925	0.250000	0.948	0.5	0.6842	0.25	1.00
Y3	4g	0.20025	0.1916	0.07841	0.980	0.19905	0.1925	0.07849	0.956	0.1994	0.1921	0.0782	1.00
O1	4g	0.3602	0.1262	0.5658	1.00	0.3627	0.1054	0.5669	1.00	0.367	0.150	0.549	1.00
O2	4g	0.5297	0.3803	0.3853	1.00	0.5260	0.3907	0.3804	1.00	0.513	0.362	0.389	1.00
O3	4g	0.1586	0.5364	0.4268	1.00	0.1744	0.5497	0.4155	1.00	0.146	0.523	0.432	1.00
O4	4g	0.3045	−0.0344	0.2829	1.00	0.3047	−0.0293	0.2886	1.00	0.290	−0.012	0.272	1.00
O5	4g	0.0514	0.0332	0.4006	1.00	0.0579	0.0467	0.4040	1.00	0.065	0.036	0.396	1.00
O6	4g	0.2222	0.4791	0.21875	1.00	0.2342	0.4719	0.2246	1.00	0.216	0.463	0.232	1.00
La1	2e	0.00000	0.7360	0.25000	0.009	0.00000	0.7389	0.25000	0.01	-	-	-	-
La2	2f	0.50000	0.6857	0.250000	0.035	0.50000	0.6892	0.25000	0.052	-	-	-	-
La3	4g	0.20025	0.1916	0.07841	0.02	0.19905	0.1925	0.07849	0.044	-	-	-	-
a, b, c (Å)		7.54419	5.33856	11.3670		7.59811	5.34229	11.3767		7.589	5.334	11.354	
α, β, γ (°)		90	104.396	90		90	104.376	90		90	104.41	90	
V (Å^3^)			446.386				447.337				445.15		
R_wp_, R_p_, χ^2^		10.22%	8.19%	6.598		12.31%	9.29%	8.416		-	-	-	

**Table 2:** The Raman peaks of all the samples and the corresponding coordination numbers (CN) are shown according to a similar formula M_2_W(Mo)O_6_. The material in parentheses, such as La_2_WO_6_, denotes that the Raman peak values equal to those in Ref. 57

No	Y_2_WO_6_	CN	Y_2_WO_6_:xLa^3+^ (x = 0.01–0.03)	CN	Y_2_WO_6_:xLa^3+^ (x = 0.04, 0.05)	CN	No	Y_2_WO_6_	CN	Y_2_WO_6_:xLa^3+^ (x = 0.01–0.03)	CN	Y_2_WO_6_:xLa^3+^ (x = 0.04, 0.05)	CN
1	-	-	934	-	934	-	22	340	6	340	6	340	6
2	834	6	834	6	834	6	23	309	6	309	6	309	6
3	-	-	798(La_2_MoO_6_)	4	-	-	24	290	6	290	6	290	6
4	-	-	773(La_2_MoO_6_)	4	773(La_2_MoO_6_)	4	25	282	6	282	6	282	6
5	707	6	714	6	707	6	26	270	6	270	6	270	6
6	694	6	694	6	694	6	27	253	6	253	6	253	6
7	668	6	668	6	668	6	28	238	6	238	6	238	6
8	645	6	645	6	645	6	29	225	6	225	6	225	6
9	621	6	621	6	621	6	30	215	6	215	6	215	6
10	596	6	596	6	596	6	31	-	-	207(Nd_2_MoO_6_)	4	207(Nd_2_MoO_6_)	4
11	550	6	550	6	550	6	32	199	6	199	6	199	6
12	521	6	521	6	521	6	33	192	6	189	6	189	6
13	501	6	501	6	501	6	34	178	6	178	6	178	6
14	471	6	466	6	466	6	35	170(Sm_2_WO_6_)	5	-	-	-	-
15	446	6	446	6	446	6	36	155(Sm_2_WO_6_)	5	156(Sm_2_WO_6_)	5	155(Sm_2_WO_6_)	5
16	426	6	426	6	426	6	37	142	6	142	6	142	6
17	394	6	394	6	394	6	38	127	6	127	6	127	6
18	-	-	381(Bi_2_MoO_6_)	4	381(Bi_2_MoO_6_)	4	39	118	6	118	6	118	6
19	375	6	-	-	-	-	40	-	-	113	6	-	-
20	364	6	362	6	362	6	41	104	6	104	6	104	6
21	354	6	354	6	354	6							

**Table 3:** The k point (in the left of parentheses) and energy values (in the right of parentheses) of La_Y1_ + V_O(36)_, La_Y2_ + V_O(24)_ and La_Y3_ + V_O(14)_ supercells. Max and Min refer to Maximum and Minimum

Models	VB	Local states	CB
La_Y1_ + V_O(36)_	(0.5, 0.053) (Max)	(0, 2.03) (Min)	(0, 3.77) (Min)
		(1.0, 3.51) (Min)	
		(0.5, 3.64) (Min)	
		(0, 3.725) (Min)	
La_Y2_ + V_O(24)_	(0.5, −0.1) (Max)	(1.0, 0.017) (Max)	(0, 3.67) (Min)
		(1.0, 0.18) (Max)	
		(0, 3.025) (Min)	
		(0, 3.286) (Min)	
La_Y3_ + V_O(14)_	(0, −0.07) (Max)	(0, 0) (Max)	(1.5, 3.64) (Min)
		(0, 0.15) (Max)	
		(0.5, 0.27) (Max)	
		(0, 2.27) (Min)	
		(1.5, 3.0) (Min)	
