# Supplementary material for: Tuning oxygen vacancy photoluminescence in monoclinic Y2WO6 by selectively occupying yttrium sites using lanthanum
Source: Sci Rep. 2015 Mar 30;5:9443. doi: 10.1038/srep09443 (PMC4377636; doi:10.1038/srep09443)
Supplement: Supplementary Information [file srep09443-s1.doc]

**Supporting Information**

**Tuning oxygen vacancy photoluminescence in monoclinic Y_2_WO_6_ by selectively occupying the yttrium sites using lanthanum**

Bangfu Ding^1^, Chao Han^1^, Lirong Zheng^2^, Junying Zhang^1^, Rongming Wang^3^ & Zilong Tang^4^

^1^Key Laboratory of Micro-nano Measurement, Manipulation and Physics (Ministry of Education), Department of Physics, Beihang University, Beijing 100191, China

^2^Beijing Synchrotron Radiation Facility, Institute of high Energy Physics, Chinese Academy of Sciences, Beijing 100049, China

^3^School of Mathematics and Physics, University of Science and Technology Beijing, Beijing 100083, China

^4^State Key Laboratory of New Ceramic and Fine Processing, Tsinghua University, Beijing 100084, China

Corresponding Author: zjy@buaa.edu.cn

**Figure S1.** Rietveld refinement results of (a) Y_2_WO_6_:0.03La^3+^ and (b) Y_2_WO_6_:0.05La^3+^. The observed and calculated patterns are shown in black fork and red solid lines. The difference between the observed and calculated patterns is given in the lowest black line. The ticks indicate the positions of Bragg reflection.

**Table S1.** ΔE (total Half widths), ΔE_l_ (the low-energy side half width), ΔE_h_ (the high-energy side half width), E_max_ (peak position) for the visible emission of Y_2_WO_6_ with different La^3+^ contents.

| Concentration (x at%) | ΔE_1/2_ (nm) | ΔE_l_ (nm) | ΔE_h_ (nm) | E_max_ (nm) |
| --- | --- | --- | --- | --- |
| x=0 | 106 | 48 | 58 | 466 |
| x=1 | 102 | 45 | 57 | 479 |
| x=2 | 103 | 44 | 59 | 473 |
| x=3 | 102 | 45 | 57 | 476 |
| x=4 | 107 | 48 | 59 | 470 |
| x=5 | 107 | 50 | 57 | 465 |

**Figure S2.** The enlarged emission spectra of 4 at% and 5 at% doping samples from 1300nm to 1700nm.

**Figure S3.** Raman spectra of Y_2_WO_6_:xLa^3+^ (a) x=0, (b) x=0.01-0.03, (c) x=0.04-0.05 powders calcined at 1250^o^C under air condition.

**Figure S4.** Fourier transforms of the extended X-ray absorption fine structure (EXAFS) signals for pure and different concentration La^3+^-doped Y_2_WO_6_ samples. The inset figure shows the nearest-neighbor O atom around W atom.

**Table S2.** Attenuation factor connected to average coordination numbers of tungsten measured using synchrotron radiation.

| Y_2_WO_6_:xLa^3+^ | x=0 | x=0.01 | x=0.02 | x=0.03 | x=0.04 | x=0.05 |
| --- | --- | --- | --- | --- | --- | --- |
| W-O bond length | 1.84Å | 1.85Å | 1.83Å | 1.87Å | 1.84Å | 1.83Å |
| Attention factor | 0.787 | 0.729 | 0.748 | 0.720 | 0.710 | 0.692 |

**Table S3**. The W-O bond length for pure, La_Y1_+V_O(36)_, La_Y2_+V_O(24)_, and La_Y3_+V_O(14)_.

| (Å) | Pure | La_Y1_+V_O(36)_ | La_Y2_+V_O(24)_ | La_Y3_+V_O(14)_ |
| --- | --- | --- | --- | --- |
| W-O1 | 1.916 | 1.889 | 1.855 |  |
| W-O2 | 1.895 | 1.845 |  | 1.833 |
| W-O3 | 1.926 |  | 1.939 | 1.833 |
| W-O4 | 1.997 | 1.956 |  |  |
| W-O5 | 2.038 | 2.001 | 1.973 | 1.979 |
| W-O6 | 2.053 |  | 2.021 | 2.820 |
| Average values | 1.971 | 1.923 | 1.945 | 2.116 |

**Figure S5.** The energy band of La_Y1_+V_O(36)_ (b) La_Y2_ +V_O(24)_ and (c) La_Y3_ +V_O(14)_, respectively.


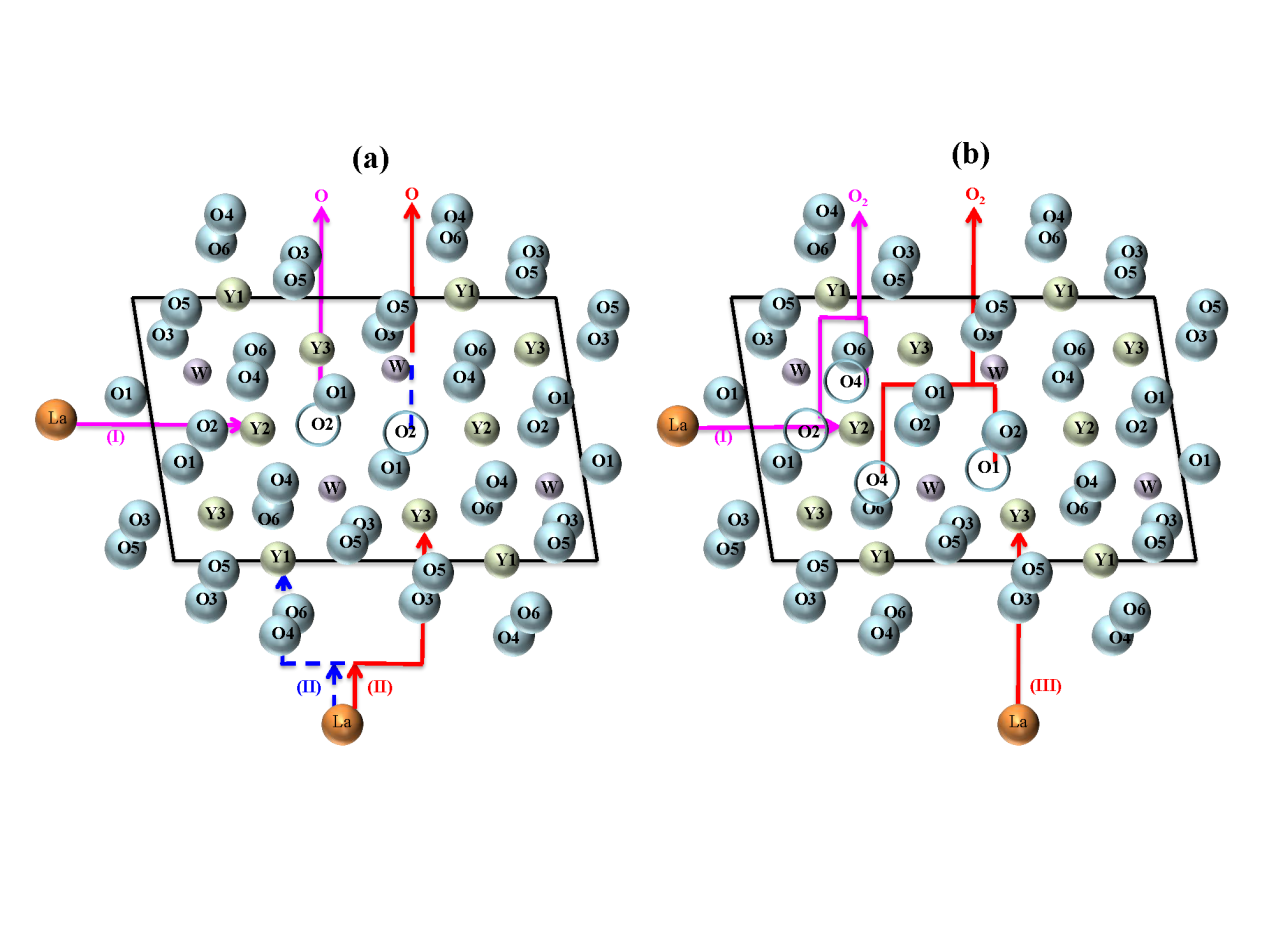


**Figure S6.** Schematic diagrams of La doping and oxygen vacancy formation. (a) When La enters into Y2 site as shown by the purple line, the nearest O2 atom escapes to form single vacancy. When La occupied Y1 or Y3 sites competitively (red solid and blue dash lines), the V_O(2)_ is also formed. (b) The twin V_O(24)_ are formed when La takes the Y2 site as displayed using purple line in low-concentration La^3+^-doped sample. At high La^3+^ doping concentration, La tend to enter Y3 sites, the red line shows the formation process of V_O(14)_.
